# Comprehensive dataset of continuously monitored hydrodynamic and physico-chemical parameters in various karst systems, France

**DOI:** 10.1038/s41597-026-07561-0

**Published:** 2026-06-17

**Authors:** Guillaume Cinkus, Raphaël Bondu, Mohammed Aliouache, Hervé Jourde, Naomi Mazzilli, Bruno Arfib, Vincent Bailly-Comte, Christelle Batiot-Guilhe, Hélène Celle, Célestine Delbart, Matthieu Fournier, David Labat, Nicolas Peyraube, Marc Steinmann, Danièle Valdès-Lao, Cécile Baudement, Sandra Béranger, Stéphane Binet, Anne Boetsch, Pascal Brunet, Simon Damien Carrière, Konstantinos Chalikakis, Jean-Baptiste Charlier, Yohann Cousquer, Christian Défarge, Joshua Ducasse, Christophe Emblanch, Juliette Fabre, Perrine Fleury, Thibaut Garin, Julien Huon, Abderrahim Jardani, Anne Johannet, Johan Jouves, Nevila Jozja, Lucile Justy, Bernard Ladouche, Thierry Lamarque, Roland Lastennet, Véronique Léonardi, Olivier Lobry, Guillaume Lorette, Christophe Loup, Pierre Marchand, Louis Manière, Jean-Christophe Maréchal, Lucie Martin, Rémi Muller, Nicolas Massei, Fabien Naessens, Chloé Ollivier, Anne Probst, Jean-Luc Seidel, Leïla Serene, Vianney Sivelle, Alexandre Zappelli

**Affiliations:** 1 UMR EMMAH, Université d’Avignon, INRAE, F-84000 Avignon, France; 2 HSM, Univ Montpellier, CNRS, IRD, Montpellier, France; 3 University Rouen Normandie, UNICAEN, CNRS, M2C UMR 6143 Rouen, France; 4 Aix Marseille Univ., CNRS, IRD, INRAE, CEREGE, Aix-en-Provence, France; 5 BRGM – French Geological Survey, F-34000 Montpellier, France; 6 G-EAU, Univ Montpellier, AgroParisTech, BRGM, CIRAD, INRAE, Institut Agro, IRD, Montpellier, France; 7 UMR 6249 Chrono-environnement, Université Marie et Louis Pasteur, 25000 Besançon, France; 8 Faculté des Sciences et Techniques, Université de Tours, EA 6293, Géo‐Hydrosystèmes Continentaux, Tours, France; 9 Université de Toulouse, Géosciences Environnement Toulouse 14 avenue Edouard Belin, 31400 Toulouse, France; 10 University of Bordeaux, I2M6GCE, CNRS 5295, 33 405 Talence, France; 11 Sorbonne Université, UMR SU CNRS EPHE 7619 METIS, Paris, France; 12 BRGM, F-31520 Ramonville-Saint-Agne, France; 13 Long-Term Socio-Ecological Research, Zone Atelier Pyrénées-Garonne, CNRS, Université de Toulouse, Campus ENSAT, F-31326 Castanet Tolosan, France; 14 ISTO, UMR 7327, Univ Orléans, CNRS, BRGM, OSUC, F-45071 Orléans, France Montpellier France; 15 UAR 3245, OSU THETA, Université Marie et Louis Pasteur, 25000 Besançon, France; 16 Institut des Sciences de la Terre d’Orléans, UMR 7327 Université d’Orléans/CNRS-INSU/BRGM, Orléans, France; 17 Cellule R&D CETRAHE, Université d’Orléans, Orléans, France; 18 UAR 3282 EMERO, University of Montpellier, CNRS, IRD FR, Montpellier, France; 19 HSM, Univ Montpellier, IMT Mines Ales, CNRS, IRD, Ales, France; 20 CENOTE, Nimes, France; 21 Spélé-H20, Six-Fours, France; 22 Université de Toulouse, Toulouse INP, CNRS, IRD, CRBE, Toulouse, France

## Abstract

Karst aquifers are a crucial source of water, supplying approximately 10% of the global population and often serving as the sole water resource in certain regions. These aquifers are characterized by highly heterogeneous flow dynamics and exhibit significant temporal variability in both hydrodynamic and physico-chemical conditions. Continuous monitoring of these parameters is essential for advancing our understanding of karst aquifer functioning; however, comprehensive, high-frequency datasets remain limited. We present a comprehensive dataset covering 13 karst springs monitored across nine observatories of the French Karst National Observatory Service (SNO KARST), spanning various hydroclimatic regions (oceanic, mountainous, Mediterranean). The SNO KARST aims to strengthen knowledge-sharing and to promote cross-disciplinary research on karst systems at the national scale. The dataset includes: (1) hydrodynamic data (water level, discharge), and (2) physico-chemical data (water temperature, electric conductivity, pH, dissolved oxygen, turbidity, Total Organic Carbon (TOC), Dissolved Organic Carbon (DOC), nitrate, and organic matter fluorescence). Spanning over a decade of continuous monitoring, such a dataset is required for the analysis of the hydrological and physico-chemical dynamics of karst aquifers, the assessment of their vulnerability to pollution and climate change, and the modeling of hydrodynamic and hydrochemical variables, ultimately aiming to improve the management and preservation of these critical water resources in contrasted contexts.

## Background & Summary

Karst regions cover approximately 15.2% of the Earth’s ice-free continental surface and are densely populated, with about 16.5% of the global population living in such areas as of 2015^1^. Karst aquifers are a vital source of freshwater, particularly in regions like the Mediterranean, where they provide, on average, about 15% of domestic water^2^ and can supply up to 90% in some areas^3^. Their broad geographic distribution and essential role in meeting water demands make them a critical component in the sustainable management of water resources. Karst systems are highly heterogeneous, with complex networks of conduits, fractures and specific geomorphological features that give rise to spatially and temporally variable hydrological processes, posing significant challenges to scientific understanding and effective resource management. They often exhibit highly dynamic, non-linear responses to external forcing, making them particularly vulnerable to contamination^2,4^. Coastal karst areas face additional risks from seawater intrusion^5,6^, especially with the overexploitation of water resource^1^. The combined effects of climate change and increasing human activity are expected to intensify these vulnerabilities, further compromising water quality and availability in karst regions^3,7–9^.

Understanding and predicting the hydrological functioning of karst systems requires a variety of complementary approaches and comprehensive datasets. Datasets like WoKaS^10^, WOKAM^11^ or MEDKAM^12^ have been instrumental in (i) compiling comprehensive information on karst regions worldwide, (ii) facilitating large-scale research, and (iii) advancing the understanding of karst systems at a global scale. However, there remains a need for more detailed, high-resolution and continuously monitored data covering a wide range of environmental variables. Indeed, such data are essential for enhancing the understanding of the spatiotemporal variability of karst systems^13^, improving the development and applicability of hydrological and hydrogeochemical models, and enabling more comprehensive comparisons across different karst regions at both regional and global scales.

The French Karst National Observatory Service (SNO KARST)^14^, as a part of the national OZCAR infrastructure^15^, addresses this need by providing a national dataset that offers continuous, high-resolution monitoring of hydrological and physico-chemical variables of karst aquifers in various hydroclimatic contexts. This dataset includes key parameters such as groundwater level, spring discharge, temperature, electrical conductivity, pH, dissolved oxygen, turbidity, total organic carbon (TOC), dissolved organic carbon (DOC), nitrate (NO_3_^-^), and organic matter (OM) fluorescence. Data are collected from nine well-established karst observatories across France, each of which monitors one or more karst systems, ensuring high data quality and regular updates to the database^14^. Each observatory encompasses karst systems with distinct characteristics depending on their location and karst morphology (e.g. Mediterranean, mountain, chalk, or edge of sedimentary basin). This wide range of conditions increases the relevance and applicability of the dataset for comparative studies and hydrological modeling, by (i) providing an overview of hydrological functioning across contrasting geological and climatic contexts, and (ii) favorising the development of interoperable tools. The dataset has already supported several integrative studies and holds considerable potential for developing transferable methodologies applicable to other karst systems ^e.g.^ ^9,16–21^, making it a valuable tool for advancing research in karst hydrology. Furthermore, the long-term monitoring, with data spanning several decades, is particularly suited for evaluating the impact of climate change on karst water resources. This includes the Mediterranean region, which is recognized as a climate change hotspot^22^ as well as mountainous regions^23^. A comprehensive list of all research associated with SNO KARST and its dataset is available through the HAL Collection at: https://hal.science/SNO-KARST/.

## Methods

### Site description and data acquisition

**Fig. 1:**
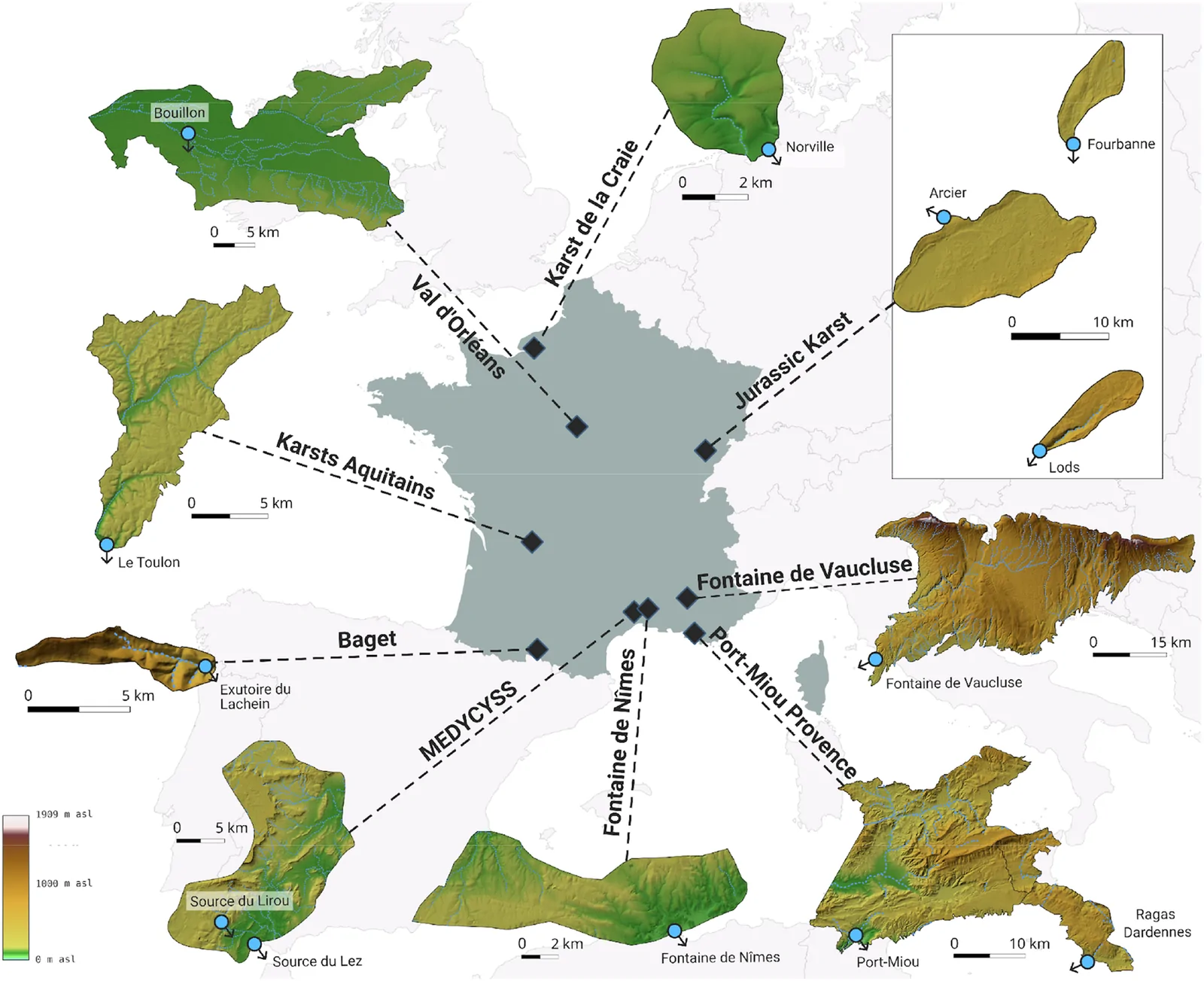
Location and extent of the SNO KARST observatories and monitoring stations.

The SNO KARST network consists of nine observatories distributed across diverse regions of France (Fig. 1), reflecting a wide range of environmental characteristics and human pressures. Each observatory includes one or more study sites, with the main instrumented site(s) typically focused on significant and representative karst outlet(s) within the region. The SNO KARST also includes three partner observatories: the Causses du Quercy Regional Park, the Grands Causses Natural Regional Park, and the Cévennes National Park. The network is specifically designed for high-frequency, continuous monitoring of hydrodynamic and physico-chemical variables, which is essential for capturing the rapid and dynamic responses typical of karst systems. Continuous measurements common to all sites include water level, temperature, and electrical conductivity. However, variation in local conditions and site-specific scientific questions determine the additional parameters that are continuously monitored. Table 1 shows the main characteristics of the observatories and their associated karst springs. Table 2 details the data monitored at each site. Altitudes are reported in meters above sea level.

**Table 1:** Main characteristics of the SNO KARST observatories and associated karst springs.

Observatory	Climate	Land use	Altitude [m asl]	Main spring	Catchment area [km^2^]	Monitoring period
Baget	Temperate oceanic, Mountain	Light agriculture	min: 498 avg: 923 max: 1417	Exutoire du Lachein	13	1968–2025
Fontaine de Nîmes	Mediterranean	Intense urban	min: 51 avg: 98 max: 200	Fontaine de Nîmes	75	1998–2025
Fontaine de Vaucluse	Mediterranean, Mountain	Moderate agriculture	min: 84 avg: 880 max: 1912	Fontaine de Vaucluse	1160	2012–2025
Jurassic Karst	Mountain, Continental, Temperate oceanic	Moderate agriculture	min: 280 avg: 400 max: 750	Arcier	100	2020–2025
				Fourbanne	30	2014–2025
				Lods	35	2014–2025
Karsts Aquitains	Continental, Temperate oceanic	Moderate agriculture	min: 79 avg: 208 max: 250	Le Toulon	100	2015–2025
Karst de la Craie	Temperate oceanic	Intensive agriculture	min: 5 avg: 90 max: 150	Norville	10	2012–2025
MEDYCYSS	Mediterranean	Moderate agriculture	min: 65 avg: 310 max: 658	Source du Lez	240	2008–2023
				Source du Lirou	240	2008–2023
Port-Miou Provence	Mediterranean	Moderate urban, light agriculture	min: 0 avg: 424 max: 1148	Port-Miou	400	2010–2025
				Ragas Dardennes	70	2012–2025
Val d’Orléans	Temperate oceanic	Moderate urban, intensive agriculture	min: 88 avg: 100 max: 130	Bouillon	284	2014–2025

**Table 2:** High-frequency monitored data by main spring. One asterisk (*) indicates a different datasource.

Observatory	Main spring	Hydrometric		Physico-chemical							
		Water level	Discharge	T	EC	pH	O_2_	Turbidity	TOC/DOC	NO_3_	OM Fluorescence
Baget	Exutoire du Lachein		×*	×	×						
Fontaine de Nîmes	Fontaine de Nîmes	×	×	×	×		×	×		×	×
Fontaine de Vaucluse	Fontaine de Vaucluse	×*	×*	×	×						×
Jurassic Karst	Arcier	×	×	×	×			×			
	Fourbanne	×	×	×	×			×	×	×	
	Lods	×	×	×	×			×	×	×	
Karsts Aquitains	Le Toulon		×	×		×	×	×			
Karst de la Craie	Norville	×	×	×	×			×			
MEDYCYSS	Source du Lez	×	×	×	×		×	×			×
	Source du Lirou	×		×	×						
Port-Miou Provence	Port-Miou	×	×	×				×			×
	Ragas Dardennes	×		×	×						
Val d’Orléans	Bouillon	×		×	×		×	×			

#### Baget

The Baget hydrosystem is located in the northern Pyrenees and covers an area of approximately 13 km^2^. The catchment is predominantly composed of calcareous lithologies from the Jurassic and Lower Cretaceous, forming a southward-sloping limestone plateau with a drainage system oriented west to east. A substantial outcrop of relatively impermeable black flysch partially covers the south-facing slope, contributing to the development of a binary karst system. The catchment elevation ranges from 498 m at the outlet to 1417 m at the Tuc de Graué, with an average of 923 m. The vegetation is predominantly a fir-beech forest, with agricultural land accounting for only 3% of the recharge area^24^. The Baget hydrosystem experiences an Atlantic oceanic climate with a clear mountainous influence, characterized by a mean annual air temperature of 12.3 °C and an average yearly rainfall of approximately 1700 mm^24–26^. The average flow of the Baget (1968–2025 period) is estimated at 0.45 m^3^ s^−1^ for a minimum observed flow of around 0.03 m^3^ s^−1^ ^27^. This site has been continuously monitored for hydrometric data, with uninterrupted discharge at a daily time step since 1968, at an hourly to half-hourly time step since 1996, and at a 15-minutes time step since 2019. The Lachein outlet is equipped with a CTD probe that allows precise monitoring of temperature, conductivity, and water level variations at 15 min intervals^28^. Baget is a key site for long-term trends analysis on hydrodynamics and hydrochemistry^16,25,27,29^ as well as for studying short-term response of the karst systems based on artificial tracer tests^30–32^ and flood event analysis^28,33^. It represents a typical example of the natural functioning of a mountainous karst system, with minimal human influence (managed forest with clear-cut areas and limited pastures).

#### Fontaine de Nîmes

The Fontaine de Nîmes spring, located in the city of Nîmes in southeastern France, is the main outlet of a karst system of approximately 55 km^2^ that extends into the garrigue area surrounding Nîmes. A well-developed karst network, primarily formed in Hauterivian (Cretaceous) limestone, includes an unsaturated zone up to 10 meters thick and a saturated zone extending several tens of meters^34^. Recharge occurs primarily through diffuse infiltration and swallow holes (unary karst). The elevation of the catchment is relatively low, ranging from 51 m at the Fontaine de Nîmes spring to nearly 200 m in the garrigue area. The climate is Mediterranean, with an average temperature of 15.2 °C and annual precipitation of 740 mm^34^, but with Mediterranean and “Cevenol” rainfall events that can bring several hundred mm of rain within a few hours. The southern part of the catchment is highly urbanized, while the northern part is covered with natural Mediterranean vegetation (garrigue). Since 1998, the discharge of the Fontaine de Nîmes spring ranges from 0.01 to 18 m^3^ s^−1^, with an average flow (1998–2025 period) of approximately 0.53 m^3^ s^−1^, while several intermittent springs flow only during flash floods^35,36^. Hydrodynamic and hydrochemical parameters are monitored via a sinkhole – Aven Mazauric – located a few meters upstream from the outlet on the main drain. Water level monitoring at an hourly time step was implemented in 1998. The measurement setup was complemented by a temperature and electrical conductivity sensor starting in 2004. Since 2014, turbidity and OM fluorescence have been monitored using fluorescence measurements, while nitrate concentrations have been monitored using absorbance measurements since 2019. More recently (2024), a dissolved oxygen probe was installed to better understand the drivers of anoxic periods at the outlet. The Fontaine de Nîmes observatory is a strategic research site for studying the contribution of groundwater to karst flash floods and has led to significant advancements in understanding their genesis and attenuation in Mediterranean karst environments^37^. Current research also uses the strong anthropogenic signature of this urban karst system to (i) describe the origin and transfer processes of water^38^, and (ii) develop innovative approaches for the early detection of groundwater contamination.

#### Fontaine de Vaucluse

The Fontaine de Vaucluse hydrosystem, located 40 km east of Avignon in southeastern France, is the largest karst system monitored within the SNO Karst network, with a catchment area of approximately 1160 km^2^. The aquifer consists primarily of a thick sequence of Cretaceous limestones, with an unsaturated zone averaging 800 m^39,40^. The conduit system extends to a depth of -204 m below sea level, a characteristic feature of “Vauclusian” systems, where the drainage network develops beneath the outlet level. The catchment spans a wide range of elevations, from 84 m at the Fontaine de Vaucluse spring to 1912 m at the summit of Mont Ventoux, with an average of 880 m, as the main part of the catchment corresponds to highlands^40^. The recharge area is predominantly natural (84%), with agricultural areas (15%) and minimal urbanization ( < 1%)^39^. It is primarily composed of hardwood, coniferous, and mixed forests, with smaller areas of scrublands, pastures, and scattered rocky outcrops. There is no permanent hydrological network. The catchment is subject to a Mediterranean climate with an average annual temperature of 10 °C and an average annual precipitation of 960 mm^40^.

The observatory’s long-term monitoring includes hydrodynamic and hydrochemical data collection from the Fontaine de Vaucluse spring. Water level and discharge have been continuously monitored at a daily time step at the spring since 1968 and at an hourly time step 400 m downstream from the spring since 2003. Daily flows from this last record (2003–2025 period) range from 3.15 m^3^ s^−1^ to 81.6 m^3^ s^−1^, with an average of 15.3 m^3^ s^−1^, making the Fontaine de Vaucluse one of the largest karst spring in Europe. Water temperature and conductivity have been monitored since 2013 with a CTD probe located 200 m downstream from the spring. The large size and complexity of the hydrosystem offer valuable insights into karst flow dynamics, variability in depositional environments, and processes of fracturing and karstification, making it a key observatory for multidisciplinary research.

#### Jurassic Karst

The Jurassic Karst Observatory comprises three monitoring sites in the Jura Mountains, each situated in distinct catchments with specific hydrogeological conditions: Fourbanne, Lods, and Arcier. The karst aquifers in the region are primarily developed in Middle to Upper Jurassic and locally in Lower Cretaceous limestones^41^. The region has a temperate climate shaped by oceanic, continental, and mountainous influences, characterized by significant temperature fluctuations and snowfall at higher elevations. Land use is primarily composed of forests, permanent grasslands, and a mix of crops and temporary grasslands. Agriculture is largely centered around dairy cattle farming. The Jurassic Karst Observatory provides a unique opportunity to study how karst systems respond to climatic gradients and land-use impacts, while offering key insights into broader biogeochemical and hydrological processes in mountain karst environments. The following paragraphs provide a more detailed description of the three monitoring sites.

The Fourbanne site is located in the Doubs valley 20 km northeast of the city of Besançon. The Fourbanne catchment covers about 30 km^2^, ranging in elevation from 260 m at the Fourbanne spring, its sole outlet, to a maximum of about 400 m. The karst aquifer is developed in tabular Middle Jurassic limestones and is mainly recharged by allogenic recharge through sinkholes^42^. The average annual precipitation ranges between 1200 and 1300 mm, the average temperature is between 10 and 11 °C^41^ and the average discharge of the Fourbanne spring is 0.81 m^3^ s^−1^ (2014–2025 period). Since 2014, the Fourbanne spring has been monitored every 30 minutes for hydrodynamic and physico-chemical parameters, including temperature and conductivity using a multiparameter probe; and DOC/TOC, turbidity, and nitrate concentrations using an UV-Vis absorbance probe. The Fourbanne site is invaluable for studying the role of physico-chemical factors in understanding the functioning of karst hydrosystems, as well as the contamination of these aquifers by nitrate of agricultural origin.

The Lods site is located 25 km southeast of Besançon and encompasses a catchment area of approximately 35 km^2^. The karst aquifer is developed in Middle and Upper Jurassic limestone and, unlike the Fourbanne site, is primarily recharged through diffuse infiltration. The catchment has two outlets at its southwestern end (the Grand Bief and Truite d’Or springs), both situated in the village of Lods in the Loue Valley at an altitude of 360 m^42^. With a maximum elevation of 960 m, the average annual precipitation is higher than at the Fourbanne site, ranging from 1400 to 1500 mm, and the average annual temperature is cooler, around 9–10 °C^41^. The discharge of the Grand Bief main spring is 1.33 m^3^ s^−1^ (2013–2025). The Grand Bief spring in Lods has been monitored every 30 minutes since 2013 for hydrodynamic parameters, temperature, and conductivity using a multiparameter probe, and since 2015 for turbidity, TOC/DOC, and nitrate using an UV-Vis absorbance probe. Similar to the Fourbanne site, but with a different hydrogeological functioning, the Lods site is important for studying the link between physico-chemical parameters and the functioning of karst aquifers, as well as the fate of nitrates in karst waters.

The Arcier site is located 10 km northeast of Besançon and covers an area of around 105 km^2^. The Arcier hydrosystem consists of a multilayered aquifer with two permeable layers constituted by the Upper and Middle Jurassic limestones and separated by a 50 m thick low-permeability layer of Oxfordian marls, with the basement of the lower aquifer formed by impervious Lower Jurassic marls^43^. The Arcier catchment receives about 1150 mm of annual precipitation and has an average temperature of 11.4 °C^44^. The Arcier spring, the main outlet of the system, has a discharge rate ranging from 0.19 to 5.88 m^3^ s^−1^, with a mean annual flow of 0.70 m^3^ s^−1^ (2020–2025 period). The Arcier spring has been monitored every 15 minutes since late 2020 for hydrodynamic and physico-chemical parameters, including temperature, pH, conductivity, and turbidity. Recently equipped, it is an important site to better understand complex flows in a multilayered karst aquifer by combining hydrostructural, geophysical (ERT, gravimetry), hydrodynamic, microbiological and hydrochemical approaches. In addition, the Arcier karst system encompasses major socio-economic challenges, as it is a critical water resource for the supply of the city of Besançon (121000 inhabitants).

#### Karsts Aquitains

The main monitoring site of the Karsts Aquitains Observatory is the Toulon karst system, which drains a catchment of about 100 km^2^ in southwestern France. Located in the city of Périgueux, the Toulon Springs – l’Abîme and le Cluzeau – are the main perennial outlets of the hydrosystem. The originality of the Toulon hydrosystem lies in its location at the edge of sedimentary basins, which entails complex groundwater dynamics. The Toulon springs are the outlet of two multilayered aquifers comprising an Upper Cretaceous limestone aquifer and an Upper Jurassic dolomitic limestone aquifer, separated by impermeable Cenomanian marls^45^. The aquifer is recharged both diffusely and through concentrated pathways, such as sinkholes and the losses of temporary streams, which activate only after heavy rainfall^46^. The Toulon springs (l’Abîme) is located at an elevation of 83 m, while the recharge area on the plateau lies primarily between 150 and 200 m. The climate is temperate oceanic with an average annual temperature of 12.5 °C and the average annual precipitation varies around 800 mm^45^. Land use is primarily forestry (~50%) and agriculture (~40%), mainly cultivating wheat, corn, sunflower, and colza, while urban areas account for less than 10%^46^. The Toulon Springs have a mean annual daily discharge of 0.45 m^3^ s^−1^ (2005–2025 period), rising to 1 m^3^ s^−1^ during rainfall events^45^. The monitoring data includes hydrodynamic parameters (Suez) and a wide range of physico-chemical parameters, such as temperature, conductivity, pH and dissolved oxygen measured with a multiparameter probe, along with turbidity concentrations monitored using an absorbance probe. Physico-chemical monitoring is conducted at 30-minute intervals, while discharge data are available every 24 hours. The Toulon hydrosystem is particularly important for research for studying the flow and chemistry dynamics in complex multilayered aquifers. They also play a role as the primary water resource for the city of Périgueux, making it a key location for studying groundwater vulnerability and contaminant transport.

#### Karst de la craie

The Karst de la Craie Observatory is located in the northern part of France, and its main monitoring site is the Norville site. The Norville karst system is located on the north bank of the Seine River, approximately 40 km upstream from its estuary, between the cities of Rouen and Le Havre. This is a chalk aquifer typical of the Upper Normandy region, with a clay-with-flint layer – formed by chalk weathering – overlying the aquifer and occasionally capped by Quaternary silts^47,48^. Swallow holes cut through the clay and chalk, allowing rapid water infiltration into the karst system. In the chalk aquifer, groundwater flows in two ways: rapid drainage through the karstic network, and a slower drainage through the micro-fissured porosity^49^. The Norville study site is a sinkhole-spring system. Upstream, the Bebec creek drains a small watershed of approximately 10 km^2^, before infiltrating into the ground through a fault^50^. Downstream, at the bottom of the chalk cliffs, the perennial Hannetot Spring overflows at the contact between chalk and the fine semi-permeable alluvial deposits of the Seine Valley^48^. The catchment exhibits a marked elevation gradient, ranging from 150 m at its headwaters to 5 m at the spring. The catchment is almost entirely dedicated to agriculture – primarily wheat and corn cultivation, along with grazing for dairy farming^47,51^ – except in the steepest areas, which are covered by deciduous forests. The climate is strongly oceanic, with average temperatures ranging from 10 to 12 °C and annual precipitation varying between 600 and 1100 mm^48^. The discharge of the Norville spring ranges from 0 to 0.1 m^3^ s^−1^, with an average flow of 0.04 m^3^ s^−1^ (2013–2025 period). It is monitored every 15 minutes for hydrodynamic and physico-chemical parameters, including temperature, conductivity, and turbidity. The Karst de la Craie Observatory is a key site for studying hydrodynamic processes in chalk karst aquifers. This is due to its simple geomorphological setting (a single karstic conduit) and its fast hydrodynamic response (within one day), which is controlled by rainfall and the Seine water level, which exhibits tidal variations rising up 6 m between low and high tide. It has made significant contributions to the interpretation of artificial tracer experiments and to the understanding of the mechanisms governing the transport of suspended solids and turbidity dynamics within these systems.

#### MEDYCYSS

The MEDYCYSS observatory (Multi scalE observatory of FlooD dYnamiCs and hYdrodynamicS in karSt) covers several karst systems in the Montpellier region, with the Lez hydrosystem being the most significant and most extensively monitored. The Lez spring, located 15 km north of Montpellier, is the main perennial outlet of the Lez karst system, which also overflows at temporary springs, with the Lirou spring being the most important. The Lez aquifer is primarily composed of Upper Jurassic and Early Cretaceous limestone. It is overlain by Lower Jurassic marls and, in some areas, covered by a sequence of Early Cretaceous marls and marly limestone^52^. This relatively impermeable marl cover limits the effective recharge area to approximately 150 km^2^, mostly where the aquifer limestone directly outcrops, within a catchment estimated at around 250 km^2^ ^53^. The aquifer shows a high degree of karstification, developed from the Middle Cretaceous to the present, with the Lez spring classified as a Vaucluse-type spring. Recharge mainly occurs through rainwater infiltration over limestone outcrops, with additional recharge via fractures and sinkholes, particularly along major faults. The catchment ranges from 65 m at Lez spring to 658 m at the summit of Pic Saint-Loup, with most of the recharge area lying between 100 and 250 m in elevation. The temporary overflow Lirou spring is located at an elevation of 90 m and only activates during high flow periods. The climate is Mediterranean, with a mean annual temperature of 14 °C and an average annual rainfall of around 940 mm^54^. However, annual precipitation varies significantly due to intense Mediterranean rainfall events, ranging from less than 600 mm in dry years to over 1 500 mm in wet years^52^. The mean annual natural discharge at the Lez spring is around 0.72 m^3^ s^−1^ (2008–2025 period) with maximum discharge of about 12 m^3^ s^−1^ and a mean pumped flow rate of 1.02 m^3^ s^−1^. Indeed, groundwater is extracted from the main karst conduit near the natural Lez Spring to supply water to approximately 350000 residents of the Montpellier metropolitan area. As a consequence, spring discharge often drops to zero when pumping exceeds the natural flow during low-flow periods. Only a reserved flow of 0.23 m^3^ s^−1^ is released into the Lez river (0.16 m^3^ s^−1^ before 2018), which also serves to monitor various physico-chemical parameters. The high-frequency data collected at the Lez spring includes water level measurements at 15 min intervals, which, when combined with pumping data, enable the estimation of the mean total flow rate exiting the karst aquifer (about 1.74 m^3^ s^−1^). The monitoring also covers a wide range of physico-chemical parameters including temperature, conductivity and dissolved oxygen, all measured using a multi-parameter probe at 60 min intervals. In addition, the data includes turbidity and the fluorescence of organic matter measured using a fluorescence probe. Data collection at the Lirou spring includes measurements of water level, temperature, and conductivity using a CTD probe, with a frequency of 15 minutes. This observatory is important for research into flood dynamics, groundwater recharge processes (quality and quantity), anthropogenic pressures on groundwater resources and the impacts of climate change on Mediterranean karst hydrology.

#### Port-Miou Provence

The Port-Miou Provence Observatory comprises two study sites located in the carbonated Provence region of southeastern France: the coastal karst system of Port-Miou and the continental karst system of Dardennes. They share a common boundary, Port-Miou discharging at the south-west of the Beausset geological unit, and Dardennes at the south-east. These hydrosystems experience a typical Mediterranean climate, with annual rainfall between 500 and 1000 mm, marked by intense spring and autumn storms ( > 100 mm/day) and prolonged summer droughts^55^. The catchment areas predominantly span a natural landscape characterized by hills, karst plateaus, and poljes^55^. The main springs emerge from vauclusian karstic conduits, which develop at depth below the outlet, in relation to paleo base levels^56^.

The Port-Miou aquifer extends over a recharge area of approximately 400 km^2^ and discharges into the Mediterranean Sea through two main submarine outlets in the Bay of Cassis, located 15 km east of Marseille: the Port-Miou and Bestouan springs. Monitoring is performed at the Port-Miou spring only. This karst aquifer is composed of Jurassic and Cretaceous limestone, dolostone and mixed siliciclastic-carbonate rocks reaching 2000–3000 m of thickness, with continuity from inland to the sea^55^. Elevations range from sea level to 1148 m at the summit of the Sainte-Baume Massif, with a median at 390 m. However, the karst conduits reach depths of over 235 m below sea level, explored by cave divers^56^. The Port-Miou and Bestouan springs are brackish, with salinity at Port-Miou spring ranging from 2 to 14 g/L^55^ calculated from electrical conductivity. Deep saline intrusion remains constant and salinity decreases due to dilution caused by increased freshwater flow^55,57^. The monitoring station is located underground at a submarine dam built in the 1970s within the saturated karst conduit, 500 m upstream of the outlet. Intended to limit seawater intrusion, the dam has proven only partially effective, as salinity remains high due to ongoing deep seawater inflow. Long-term monitoring is carried out at the Port-Miou underground station, including measurements of water level upstream and downstream the dam, temperature, conductivity, and turbidity at 5 or 15 min intervals, and some of them are averaged at daily time step. Port-Miou spring is among the largest karst springs in France. Spring’s discharge is only available on the period 2010–2014 (mean of 7.65 m^3^ s^−1^), during a period of above-average precipitation^55^. The water level upstream of the submarine dam is influenced by sea level, karst discharge and evolution of the gauging station. The water level downstream of the dam, on the other hand, depends mainly on sea level. This site is particularly valuable for research on the dynamics of saline intrusion, the interaction between freshwater and seawater, and the effects of climate change on coastal karst systems.

The Dardennes karst system extends over the Siou Blanc massif, a karst plateau located northwards from the city of Toulon. The aquifer is developed within early Cretaceous limestones and late Jurassic dolostones, with a total thickness exceeding 900 m. The plateau displays intensive karstification, which promotes rapid infiltration of rainfall and the absence of surface stream^58^. The recharge area is estimated around 70 km^2^. The median elevation is ~620 m, with the highest point at 826 m. Land cover is predominantly natural, characterized by Mediterranean garrigue vegetation. The Dardennes aquifer discharges through several perennial springs located beneath an artificial lake, which serves as a drinking water reservoir for the city of Toulon, at an elevation of ~100 m and typically submerged under 10 to 23 meters of water^58^. The remaining groundwater feeds the Las river downstream, where flash floods can occur due to urban runoff or karst overflow^59,60^. The Ragas Dardennes is a temporary overflow spring located 500 m upstream of the reservoir, with huge and rapid water level fluctuations up to 50 m in a few hours. The monitoring station has been located in the karst conduit of the Ragas Dardennes since 2012 and includes measurements of water level, temperature, and conductivity using CTD probes every 15 min. The total discharge of the aquifer through the Dardennes springs has been calculated for the period 2012–2015^58^, and ranges from 0.05 to over 30 m^3^ s^−1^. The Dardennes site is important for understanding and modeling karst hydrodynamics, particularly for flood prediction and the management of karst water resources.

#### Val d’Orléans

The Val d’Orléans Observatory is located in the Loire valley, approximately 130 km south of Paris, and focuses on the Loiret springs to the southeast of the city of Orléans. The karst aquifer is hosted within an Oligocene carbonate lacustrine deposit called the Beauce limestone with a high porosity overlain by the Quaternary alluvium of the Loire River^61^. This geological setting results in significant fluxes between the alluvial aquifer and the karst aquifer. In some areas, a clay layer is interbedded, creating confined conditions within the karst aquifer. The Loiret springs catchment extends over 284 km^2^ and is dominated by agricultural land use – primarily market gardening – along with areas of urban development^62^. However, the aquifer is primarily recharged by losses from the Loire River near the town of Jargeau, approximately 10 km upstream from Orléans^63^. It is estimated that the Loire River supplies between 80% and 96% of the karst groundwater, primarily through sinkholes in the riverbed^61,64^. Water flows through the karst network and discharges at several springs feeding the Loiret River with an estimated residence time of approximately 3.5 days. The Bouillon Spring constitutes the principal resurgence, with discharge rates ranging from 0.1 to 5 m^3^ s^−1^. The elevation gradient ranges from 88 m at the Bouillon to around 97 m at the Jargeau losses. The climate of Orléans is of the degraded oceanic type, with average annual temperatures around 11 °C and average annual precipitation around 730 mm^65^. Data collection at the Bouillon Spring involves monitoring water level and physico-chemical parameters – temperature, conductivity and dissolved oxygen at 15 min intervals with a multiparameter probe, and turbidity at the same interval using an absorbance probe. However, water level and discharge measurements are subject to uncertainty, as the course of the Loiret has been modified by a series of historical causeways constructed for mill operations, leading to a stepped base level control of the resurgence flow in the different basins^63^. The Val d’Orléans Observatory is a key site for investigating the dynamics of losses and resurgence in karst aquifers, as well as surface-groundwater interactions, utilizing both modeling and natural/artificial tracing techniques. This approach has yielded valuable insights into hydrochemical evolution, dilution, and mixing processes.

### Data processing

Each observatory is responsible for the entire process, from data acquisition to integration into the database. This task is carried out by the observatory managers and their teams and involves: (1) selecting variables for continuous monitoring based on local and historical scientific objectives, extending beyond core measurements of water level, temperature, and conductivity; (2) data acquisition, which includes the installation and maintenance of probes, as well as data retrieval; and (3) data processing and validation prior to entry into the database. The database contains three levels of data: (1) raw data, which are unprocessed data requiring appropriate transformations for use; (2) quality-controlled data, which have been validated by the observatory managers; and (3) derived data, which are calculated from other measurements (e.g., discharge derived from water levels using a rating curve). The quality-controlled data are processed and validated by the observatories teams, as this process requires expert knowledge in each karst hydrosystem. Data processing primarily consists of detecting and replacing outliers – typically caused by sudden and abrupt shifts in sensor readings, often due to external disturbances, field intervention, or electrical glitches – as well as identifying and correcting measurement drifts, which manifest as slow and progressive deviations in recorded values, primarily caused by biofouling, infrequent calibration, or sensor aging. Special care was taken during data processing to account for the highly dynamic nature of karst systems, in order to avoid the inadvertent exclusion of valid data. Periods of data gaps due to sensor interruptions or transmission failures are left unchanged and no imputation is performed for missing values, in order to preserve the raw structure of the dataset and to allow users to apply their preferred processing methods for data reconstruction. Although monitoring timeframes, measurement frequencies, and measured parameters may vary across observatories depending on local scientific objectives, continuous efforts are made to harmonize the measurement of similar variables among teams. This is particularly the case for CTD probe installation protocols, as well as for the correction of outliers and sensor drift in UV–visible absorbance and fluorescence measurements, with ongoing work to better account for the influence of turbidity. These efforts aim to improve the comparability of datasets across observatories. The units used to each continuously monitored parameter are summarized in Table 3.

**Table 3:** Units of continuously monitored hydrodynamic and physico-chemical parameters.

Parameter	Unit
Groundwater Level	m
Discharge (derived product)	m^3^/s
Temperature	°C
Conductivity	µS/cm
pH	pH
Turbidity	NTU
TOC/DOC	mg/L
NO_3_	mg/L
OM fluorescence	mV

## Data Records

Datasets are stored in the OSU OREME repository as a relational database using the PostgreSQL Database Management System associated with the PostGIS spatial extension. The database relational schema allows to include rich metadata information that describe the stored datasets (station, instruments, methods, persons, etc). Metadata are based as much as possible on semantic artefacts (thesaurus, repositories, etc.) to improve semantic interoperability. More generally, the database is part of the global OSU OREME information system that delivers the datasets in respect of the FAIR principles (data are Findable, Accessible, Interoperable and Reusable). The datasets are accessible through the web OREME data portal (https://data.oreme.org) that partly relies on standard software (GeoServer, GeoNetwork) implementing the standard protocols of the Open Geospatial Consortium (OGC). The datasets can be visualized using dynamic and interactive chart viewers. Data can also be queried through an API to enable automated and programmatic processing. This facilitates reproducible workflows and integration into data analysis pipelines. The API is a FastAPI-based web service, organized into six main resource types, each with its own router (https://api.oreme.org/snokarst/docs). All endpoints follow RESTful conventions and allow query parameters and pagination (format, page, filter fields). Default page size is 20000 items (up to 50000 items, customizable via a “size” parameter). Station responses include GeoJSON geometry when available. CSV exports use streaming responses to minimize memory usage, pagination to prevent loading entire datasets into memory and async database operations to improve concurrency. Alongside FastAPI, the API is using SQLAlchemy for database operations, GeoAlchemy for geographic data support, Pydantic for data validation and Pandas for CSV generation.

It should be noted that SNO Karst high-frequency data are not available in the ADES database (Accès aux données sur l’eau souterraine; https://ades.eaufrance.fr/), which is the French governmental repository for groundwater data. Furthermore, only daily time step data are available in the ADES repository, which represents a significant limitation for the study of reactive karst systems where variations can occur at an hourly scale or less.

The dataset is publicly available on the OSU OREME data portal^66–90^, under the “data export” section (https://data.oreme.org/observation/snokarst). It is licensed under the Licence Ouverte 2.0 (https://www.etalab.gouv.fr/wp-content/uploads/2018/11/open-licence.pdf). Table 4 provides the direct DOI links for all observatories and types of data, each leading to a prefilled form on the data portal, which includes: (i) observatory name, (ii) hydrological entity, (iii) name of the station (drop-down menu), (iv) parameter category, (v) measurement type (drop-down menu), (vi) date range (from/to), (vii) the inclusion of deprecated time series (TRUE/FALSE), and (viii) the restriction to storm event data (TRUE/FALSE). The query returns three text files which can be downloaded individually or all at once:

- A CSV data file with date and available time series as columns. It is named “snokarst_*name*_*datetime*_data.csv”, where “name” corresponds to the spring name and “datetime” to the date and time in %Y%m%d%H%M format (e.g. snokarst_toulon_202604291512.csv). The number of columns and rows is related to the available variables and the length and time step of the record. The header gives information about the name of the variables, units, time series id, name of the station and quality of the data (lines 1, 2, 3, 4 and 5, respectively). The following variables can be found depending on the site: water_level, discharge, water_temperature, conductivity, pH, dissolved_oxygen, turbidity, TOC, DOC, NO_3_, fluorescence (Table 2);
- A CSV metadata file with descriptive information about time series (station, parameter, instrument, organisations, etc.). It is named “snokarst_*name*_*datetime*_metadata.csv”, following the same naming convention as the data file. This file is mandatory to have a description of the probes and parameters measured or processed;
- A “Use conditions” PDF file with details about licensing, reference, general and specific use conditions, and the SNO KARST data policy.

**Table 4:** Available high-frequency datasets by spring and type, with corresponding DOIs.

Observatory	Main spring	Type of data	Access
Baget	Exutoire du Lachein	Hydrometric	https://doi.org/10.15148/c9cc27d4-00d0-440c-82b6-29600916a2f6#402,auto https://www.hydro.eaufrance.fr/sitehydro/O0485110/fiche
		Physico-chemical	https://doi.org/10.15148/b858689a-aaad-4e2c-8ef4-9f94e211e188#668,auto
Fontaine de Nîmes	Fontaine de Nîmes	Hydrometric	https://doi.org/10.15148/e73a5c65-2a7d-46e5-8e27-6edf5e452aeb#461,auto
		Physico-chemical	https://doi.org/10.15148/5d4514e2-61fe-46c4-aa72-316be44fd3ae#461,auto
Fontaine de Vaucluse	Fontaine de Vaucluse	Hydrometric	https://www.hydro.eaufrance.fr/sitehydro/V6155020/fiche
		Physico-chemical	https://doi.org/10.15148/fb67e0b8-4b19-4c46-875b-0e2d44ea485f#838,auto
Jurassic Karst	Arcier	Hydrometric	https://doi.org/10.15148/792c2468-d839-4ad3-8ef0-b928a6656e8c#830,auto
		Physico-chemical	https://doi.org/10.15148/bdfffa66-860c-4245-a11a-4fbbabc521e3#830,auto
	Fourbanne	Hydrometric	https://doi.org/10.15148/3b65d490-0aeb-4b30-84a5-c5d46cfa855b#406,auto
		Physico-chemical	https://doi.org/10.15148/09929cf0-6a6b-414e-b5bd-25a9b8c78d88#406,auto
		Chemical	https://doi.org/10.15148/841d3677-fddc-454c-8a71-abac2851ed6f#406,auto
	Lods	Hydrometric	https://doi.org/10.15148/a2ca20f9-cd78-4260-84d4-ceb4b1426666#407,auto
		Physico-chemical	https://doi.org/10.15148/5ebb9d5f-081a-405a-8290-ad29a084e4e9#407,auto
		Chemical	https://doi.org/10.15148/a815f209-0ca1-4240-89fd-e0d0418a6e5d#407,auto
Karst Aquitains	Le Toulon	Hydrometric	https://doi.org/10.15148/fcf00f41-f22c-47c0-beb4-031255793259#651,auto
		Physico-chemical	https://doi.org/10.15148/6c7a0db4-2515-4488-9e70-94cc4454fa8a#472,auto
Karst de la Craie	Norville	Hydrometric	https://doi.org/10.15148/15ad51cd-784c-4ef7-b547-bd3c620c0512#469,auto
		Physico-chemical	https://doi.org/10.15148/e4d0ad5d-eaab-4f9d-baf4-b34f078f89c0#469,auto
MEDYCYSS	Source du Lez	Hydrometric	https://doi.org/10.15148/cfd01a5b-b7fd-41aa-8884-84dbddac767e#241,auto
		Physico-chemical	https://doi.org/10.15148/38bd5fa8-a7e1-4900-a1f6-2d59c3f31dfe#241,auto
	Source du Lirou	Hydrometric	https://doi.org/10.15148/cfd01a5b-b7fd-41aa-8884-84dbddac767e#257,auto
		Physico-chemical	https://doi.org/10.15148/38bd5fa8-a7e1-4900-a1f6-2d59c3f31dfe#258,auto
Port-Miou Provence	Port-Miou	Hydrometric	https://doi.org/10.15148/152ea7a3-a754-4248-bd5d-2bfec1bff17d#470,auto
		Physico-chemical	https://doi.org/10.15148/a757d9f3-49f4-42a4-80c6-2a7c423fa156#470,auto
	Ragas Dardennes	Hydrometric	https://doi.org/10.15148/e3fcfeb1-e683-4bfe-b5e8-717dd04e2fb4#699,auto
		Physico-chemical	https://doi.org/10.15148/18b4d8bf-aa12-4159-92fc-23ec70d1198e#699,auto
Val d’Orléans	Bouillon	Hydrometric	https://doi.org/10.15148/ec4d084c-ed89-4cd0-946a-076bf166a150#399,auto
		Physico-chemical	https://doi.org/10.15148/cdf4f839-d6a8-4047-86fc-4f81b2295db6#399,auto

**Fig. 2:**
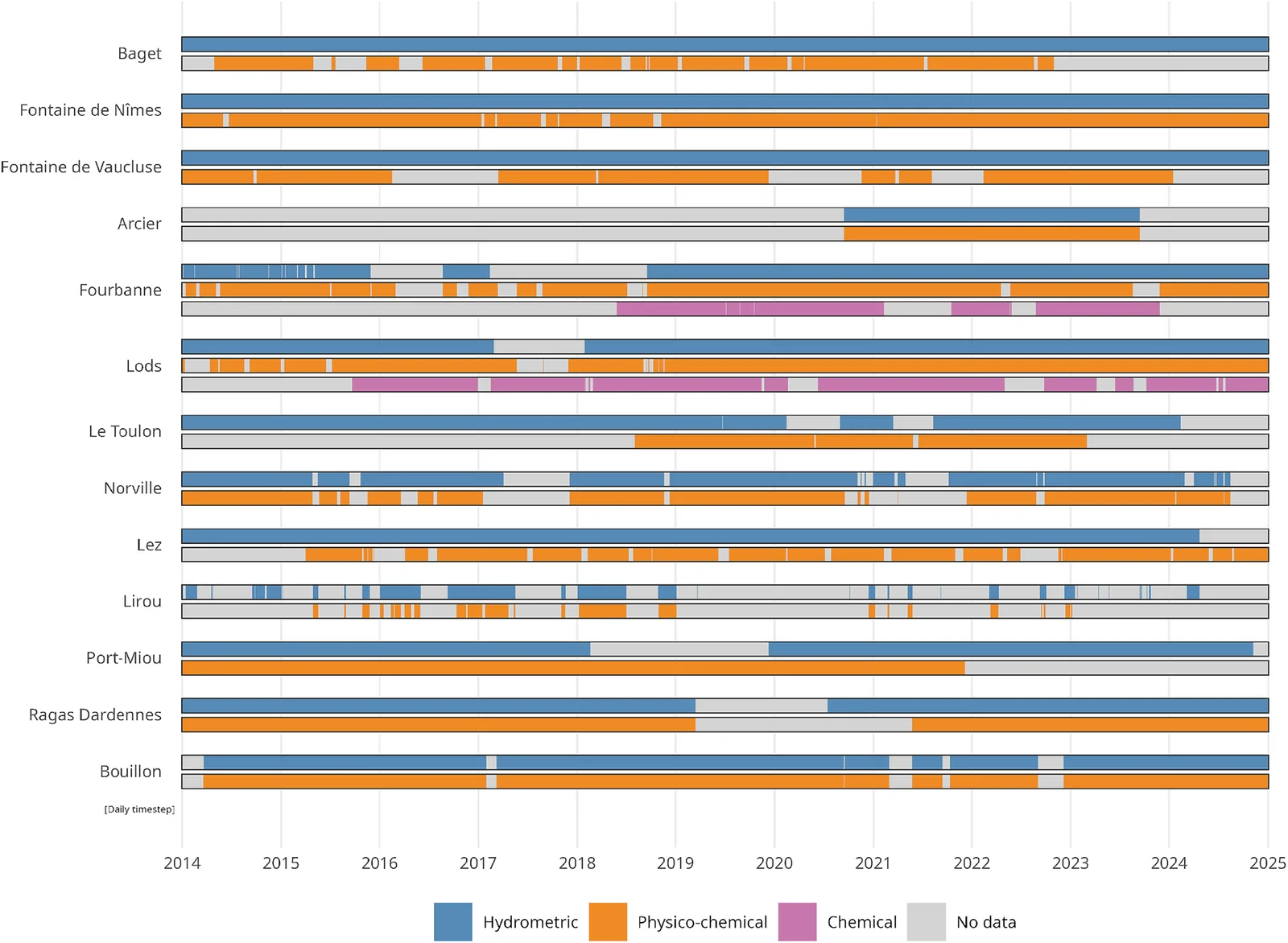
Data availability by type for each observatory since the creation of the SNO KARST (2014). The total duration is reported in Table 1.

Type of data for each site are displayed in Table 2, and greater detail about data availability can be found in Fig. 2. Variables can be available as raw data or quality-controlled data. The records are updated each year.

## Data Overview

**Fig. 3:**
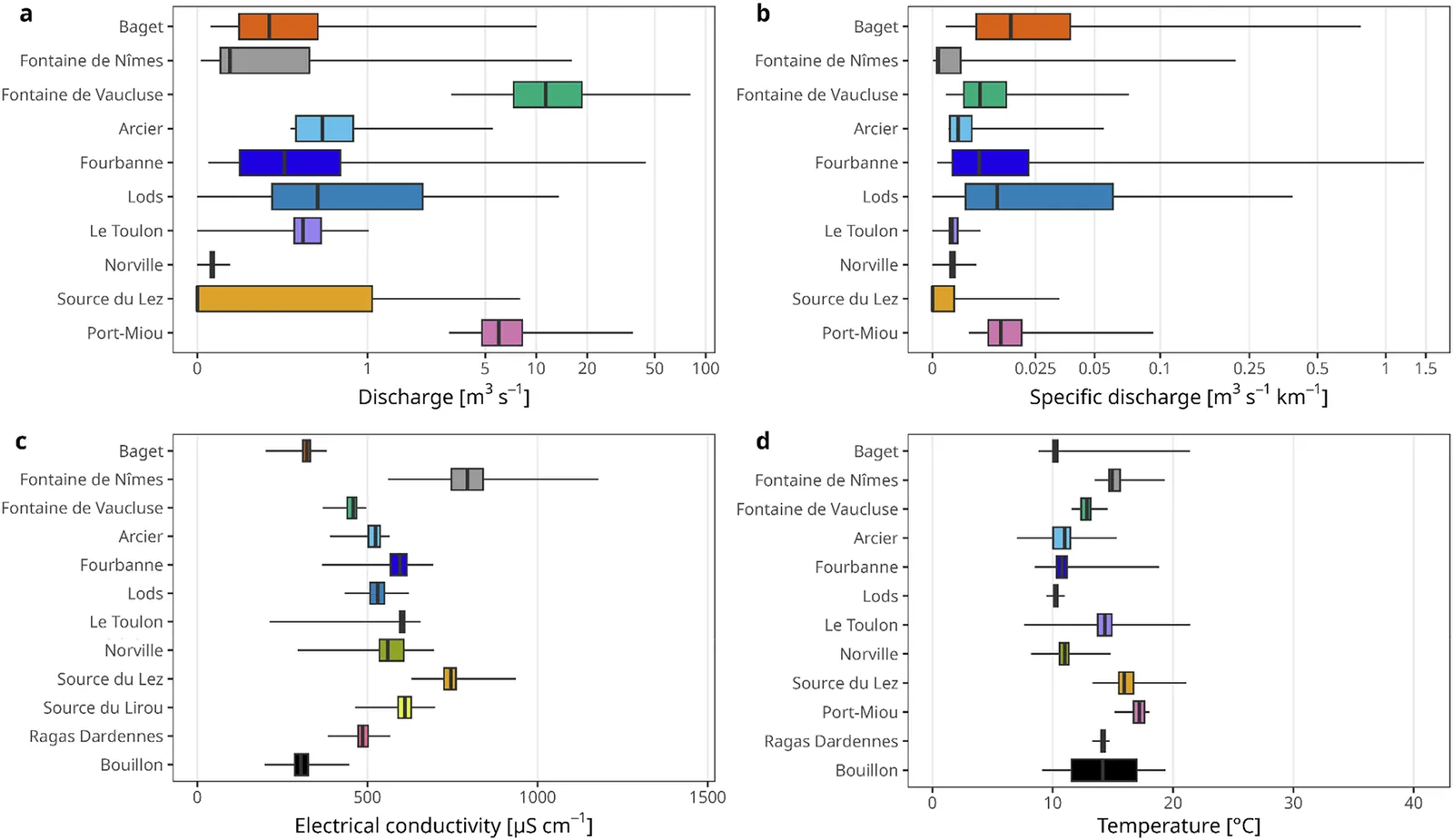
Boxplot of different variables on each station, displaying full data range (whiskers extend to minimum and maximum values). (**a**) Spring discharge; (**b**) Specific spring discharge; (**c**) Water electrical conductivity; (**d**) Water temperature.

Figure 3 presents the distribution of the values of discharge, specific discharge, electrical conductivity and temperature from the karst hydrosystems in each observatory, calculated on the basis of data availability (Fig. 2). This highlights the wide range of hydrological functioning that is typical of karst, thus reflecting the high diversity of karst systems available in the dataset.

## Technical Validation

### Instrumentation

The monitoring network comprises several types of *in situ* recording instruments. CTD probes are used to measure water level, temperature, and electrical conductivity (e.g., NKE CTD Leveline, vanEssen CTD Diver). In addition to water level, temperature, and conductivity, multiparameter probes record a range of physicochemical variables, including pH, oxidation–reduction potential (ORP), dissolved oxygen, and turbidity (e.g., *In-Situ* AquaTROLL, NKE WiMo). UV–visible absorbance probes are deployed to estimate total and dissolved organic carbon, turbidity, and nitrate concentrations (e.g., S::can spectro::lyser). Finally, fluorescence-based probes are used to quantify organic matter fluorescence and turbidity (e.g., Albillia GGUN). These instruments originate from different manufacturers, reflecting the technical choices made by the individual observatory teams. To ensure measurement accuracy, all instruments are calibrated following the manufacturers’ recommended procedures and at the suggested time intervals. Calibration procedures vary according to sensor technology. CTD probes are calibrated for pressure, temperature, and electrical conductivity using factory calibration, with periodic verification against certified reference values. Multiparameter probes, including *In-Situ* AquaTROLL and NKE WiMo instruments, are calibrated on a parameter-by-parameter basis using dedicated standards (e.g., pH buffer solutions, conductivity standards, dissolved oxygen references, and turbidity standards). UV–visible S::can spectro::lyser probes differ from these sensors in that they are factory pre-calibrated using spectral models that convert absorbance spectra into estimates of nitrate, organic carbon, and turbidity. Site-specific calibration can subsequently be performed by correlating *in situ* spectra with laboratory analyses of discrete water samples. Fluorescence-based GGUN probes are calibrated optically using clean water blanks and standard solutions with known tracer or turbidity concentrations. Rating curves for deriving flow discharge from water levels were established based on multiple measurements taken across a range of flow conditions, from low to very high flow periods. If unexpected shifts or discrepancies were observed, a secondary probe was deployed as a redundancy measure to determine whether the phenomenon was natural or due to an equipment issue. The growing adoption of remote data transmission allows for real-time monitoring and rapid detection of measurement errors caused by probe malfunctions.

### Data validation

To ensure the quality and accuracy of the data, high-frequency parameters are validated against punctual measurements conducted in the field, using (1) multiparameter field probes for temperature, conductivity, pH, and dissolved O₂; (2) portable spectrophotometers for turbidity and, in some cases, NO₃ and Cl; and (3) laboratory analyses of collected samples for NO₃, Cl and TOC/DOC. Field measurements served as a reference for data validation: any significant and consistent deviations were corrected based on the differences between the field measurements and the corresponding points in the time series. It is important to note that these corrections are performed by observatory teams, as the pronounced and unique variability of karst hydrosystems requires expert knowledge of their behavior, supported by long-term observational experience. Moreover, since discharge values are derived from the rating curve, a range of complementary methods is regularly employed to validate the discharge data, including the use of tracer dye methods, Doppler flowmeters, as well as water balance calculations. Rating curves are updated when significant discrepancies between methods are observed.

## Usage Notes

The dataset can be used for comparative studies, hydrological analyses, modelling of karst processes, and climate change impact studies, among other applications. In any study involving this dataset, it is relevant to consider the specificities of each karst system, such as anthropogenic influence, geological context, karstification, catchment area, and climatic conditions. These factors can significantly impact the hydrodynamic and physico-chemical responses of the systems, and thus be relevant to consider in any related study.

The dataset can be integrated into dedicated karst-focused software such as KarstMod^91,92^ and KarstID^93^, which are developed and freely distributed by the SNO KARST to provide robust tools for analyzing, classifying, and modelling the hydrodynamic response of karst systems. Other tools can also be found in the scientific literature, such as XLKarst^36^, V2Karst^94^ or LuKARS^95^, among others. These programs are specifically designed to handle the complexity of karst environments and offer appropriate functions for the interpretation of time series data.

To ensure a relevant analysis, it is recommended to use the lowest possible time step available when working with the dataset, as aggregating over larger intervals may conceal the rapid dynamics inherent to karst systems. While daily time steps may be appropriate for systems with a high inertia response, it could affect small-scale events in more reactive systems that have an hourly response. When conducting comparative studies across multiple systems, we recommend using consistent timeframes and time steps to ensure meaningful results. Metrics such as the coefficient of variation or cross-correlation analyses with precipitation can be valuable tools for assessing and understanding the reactivity, behavior and response times of a system.

## Data Availability

The SNO KARST time series datasets are accessible under the “data export” section of the OSU OREME data portal (https://data.oreme.org/observation/snokarst) and are each associated with an unique (https://doi.org/10.15148/c9cc27d4-00d0-440c-82b6-29600916a2f6#402,auto; https://doi.org/10.15148/b858689a-aaad-4e2c-8ef4-9f94e211e188#668,auto; https://doi.org/10.15148/e73a5c65-2a7d-46e5-8e27-6edf5e452aeb#461,auto; https://doi.org/10.15148/5d4514e2-61fe-46c4-aa72-316be44fd3ae#461,auto; https://doi.org/10.15148/fb67e0b8-4b19-4c46-875b-0e2d44ea485f#838,auto; https://doi.org/10.15148/792c2468-d839-4ad3-8ef0-b928a6656e8c#830,auto; https://doi.org/10.15148/bdfffa66-860c-4245-a11a-4fbbabc521e3#830,auto; https://doi.org/10.15148/3b65d490-0aeb-4b30-84a5-c5d46cfa855b#406,auto; https://doi.org/10.15148/09929cf0-6a6b-414e-b5bd-25a9b8c78d88#406,auto; https://doi.org/10.15148/841d3677-fddc-454c-8a71-abac2851ed6f#406,auto; https://doi.org/10.15148/a2ca20f9-cd78-4260-84d4-ceb4b1426666#407,auto; https://doi.org/10.15148/5ebb9d5f-081a-405a-8290-ad29a084e4e9#407,auto; https://doi.org/10.15148/a815f209-0ca1-4240-89fd-e0d0418a6e5d#407,auto; https://doi.org/10.15148/fcf00f41-f22c-47c0-beb4-031255793259#651,auto; https://doi.org/10.15148/6c7a0db4-2515-4488-9e70-94cc4454fa8a#472,auto; https://doi.org/10.15148/15ad51cd-784c-4ef7-b547-bd3c620c0512#469,auto; https://doi.org/10.15148/e4d0ad5d-eaab-4f9d-baf4-b34f078f89c0#469,auto; https://doi.org/10.15148/cfd01a5b-b7fd-41aa-8884-84dbddac767e#241,auto; https://doi.org/10.15148/38bd5fa8-a7e1-4900-a1f6-2d59c3f31dfe#241,auto; https://doi.org/10.15148/cfd01a5b-b7fd-41aa-8884-84dbddac767e#257,auto; https://doi.org/10.15148/38bd5fa8-a7e1-4900-a1f6-2d59c3f31dfe#258,auto; https://doi.org/10.15148/152ea7a3-a754-4248-bd5d-2bfec1bff17d#470,auto; https://doi.org/10.15148/a757d9f3-49f4-42a4-80c6-2a7c423fa156#470,auto; https://doi.org/10.15148/e3fcfeb1-e683-4bfe-b5e8-717dd04e2fb4#699,auto; https://doi.org/10.15148/18b4d8bf-aa12-4159-92fc-23ec70d1198e#699,auto; https://doi.org/10.15148/ec4d084c-ed89-4cd0-946a-076bf166a150#399,auto; https://doi.org/10.15148/cdf4f839-d6a8-4047-86fc-4f81b2295db6#399,auto). Direct DOI links for each observatory and data type are provided in Table 4.

## Code availability

No custom code can be provided as the operations are done with different tools by each observatory.

## References

[1] Goldscheider, N. *et al*. Global distribution of carbonate rocks and karst water resources. *Hydrogeol. J.* **28**, 1661–1677 (2020).

[2] Bakalowicz, M. Karst and karst groundwater resources in the Mediterranean. *Environ. Earth Sci.* **74**, 5–14 (2015).

[3] Stevanović, Z. Karst waters in potable water supply: A global scale overview. *Environ. Earth Sci.* **78**, 662 (2019).

[4] Hartmann, A. *et al*. Risk of groundwater contamination widely underestimated because of fast flow into aquifers. *Proc. Natl. Acad. Sci. USA* **118**, e2024492118 (2021). 10.1073/pnas.2024492118 PMC8158018 33972438

[5] Arfib, B., De Marsily, G. & Ganoulis, J. Locating the Zone of Saline Intrusion in a Coastal Karst Aquifer Using Springflow Data. *Ground Water* **45**, 28–35 (2007). 10.1111/j.1745-6584.2006.00252.x 17257336

[6] Pinault, J.-L., Doerfliger, N., Ladouche, B. & Bakalowicz, M. Characterizing a coastal karst aquifer using an inverse modeling approach: The saline springs of Thau, southern France: CHARACTERIZING A COASTAL KARST AQUIFER. *Water Resour. Res*. **40**, (2004).

[7] Parise, M., Gabrovsek, F., Kaufmann, G. & Ravbar, N. Recent advances in karst research: From theory to fieldwork and applications. *SP* **466**, 1–24 (2018).

[8] Giese, M., Caballero, Y., Hartmann, A. & Charlier, J.-B. Trends in long-term hydrological data from European karst areas: Insights for groundwater recharge evaluation. *Hydrol. Earth Syst. Sci.* **29**, 3037–3054 (2025).

[9] Binet, S. *et al*. Global warming and acid atmospheric deposition impacts on carbonate dissolution and CO2 fluxes in French karst hydrosystems: Evidence from hydrochemical monitoring in recent decades. *Geochimica et Cosmochimica Acta* **270**, 184–200 (2020).

[10] Olarinoye, T. *et al*. Global karst springs hydrograph dataset for research and management of the world’s fastest-flowing groundwater. *Sci. Data* **7**, 59 (2020). 10.1038/s41597-019-0346-5 PMC7033224 32080203

[11] Chen, Z. *et al*. The World Karst Aquifer Mapping project: Concept, mapping procedure and map of Europe. *Hydrogeol J* **25**, 771–785 (2017).

[12] Xanke, J. *et al*. *Mediterranean Karst Aquifer Map 1:5,000,000 (MEDKAM)* 10.25928/MEDKAM.1 (2022).

[13] Serène, L. *et al*. High-frequency hydrodynamic and hydrochemical data from karst unsaturated zone. *Data in Brief* 111812, 10.1016/j.dib.2025.111812 (2025). PMC12269470 40677258

[14] Jourde, H. *et al*. SNO KARST: A French Network of Observatories for the Multidisciplinary Study of Critical Zone Processes in Karst Watersheds and Aquifers. *Vadose Zone J* **17**, 180094 (2018).

[15] Gaillardet, J. *et al*. OZCAR: The French Network of Critical Zone Observatories. *Vadose Zone Journal* **17**, 1–24 (2018).

[16] Sivelle, V., Labat, D., Mazzilli, N., Massei, N. & Jourde, H. Dynamics of the Flow Exchanges between Matrix and Conduits in Karstified. *Watersheds at Multiple Temporal Scales. Water* **11**, 569 (2019).

[17] Lorette, G. *et al*. Tracing water perturbation using NO3-, doc, particles size determination, and bacteria: A method development for karst aquifer water quality hazard assessment. *Science of The Total Environment* **725**, 138512 (2020). 10.1016/j.scitotenv.2020.138512 32302853

[18] Cinkus, G., Mazzilli, N. & Jourde, H. Identification of relevant indicators for the assessment of karst systems hydrological functioning: Proposal of a new classification. *J. Hydrol.* **603**, 127006 (2021).

[19] Serène, L. *et al*. Transit Time index (TTi) as an adaptation of the humification index to illustrate transit time differences in karst hydrosystems: Application to the karst springs of the Fontaine de Vaucluse system (southeastern France). *Hydrol. Earth Syst. Sci.* **26**, 5035–5049 (2022).

[20] Wunsch, A. *et al*. Karst spring discharge modeling based on deep learning using spatially distributed input data. *Hydrol. Earth Syst. Sci.* **26**, 2405–2430 (2022).

[21] Grandhay, C. *et al*. Yeast Biodiversity of Karst Waters: Interest of Four Culture Media and an Improved MALDI-TOF MS Database. *Microb. Ecol.* **87**, 26 (2024). 10.1007/s00248-023-02336-1 PMC10766713 38175217

[22] Lionello, P. & Scarascia, L. The relation between climate change in the Mediterranean region and global warming. *Reg Environ Change* **18**, 1481–1493 (2018).

[23] Giese, M., Caballero, Y., Hartmann, A. & Charlier, J.-B. *Trends in long-term hydrological data from European karst areas: Insights for groundwater recharge evaluation*10.5194/egusphere-2024-2078 (2024).

[24] Richieri, B. *et al*. On the value of hydrochemical data for the interpretation of flow and transport processes in the Baget karst system, France. *Hydrogeol J* **32**, 1537–1555 (2024).

[25] Ulloa-Cedamanos, F. *et al*. A Forty-Year Karstic Critical Zone Survey (Baget Catchment, Pyrenees-France): Lithologic and Hydroclimatic Controls on Seasonal and Inter-Annual Variations of Stream Water Chemical Composition, pCO2, and Carbonate Equilibrium. *Water* **12**, 1227 (2020).

[26] Al Khoury, I. *et al*. Evaluation of precipitation products for small karst catchment hydrological modeling in data-scarce mountainous regions. *Journal of Hydrology* **645**, 132131 (2024).

[27] Labat, D. *et al*. New insights from an enhancement of hydrogeological archives of a French karst watershed over the last 50 years. *Comptes Rendus. Géoscience* **355**, 523–531 (2024).

[28] Ulloa-Cedamanos, F. *et al*. Stream Hydrochemical Response to Flood Events in a Multi-Lithological Karstic Catchment from the Pyrenees Mountains (SW France). *Water* **13**, 1818 (2021).

[29] Ulloa-Cedamanos, F. *et al*. Potential influence of landscape transition on stream water chemistry trends during the last decades in a karst catchment (Pyrenees, SW France) in a context of global environmental changes. *Ecological Indicators* **140**, 109023 (2022).

[30] Labat, D. & Mangin, A. Transfer function approach for artificial tracer test interpretation in karstic systems. *Journal of Hydrology* **529**, 866–871 (2015).

[31] Sivelle, V. & Labat, D. Short-term variations in tracer-test responses in a highly karstified watershed. *Hydrogeol J* **27**, 2061–2075 (2019).

[32] Sivelle, V., Renard, P. & Labat, D. Coupling SKS and SWMM to Solve the Inverse Problem Based on Artificial Tracer Tests in Karstic Aquifers. *Water* **12**, 1139 (2020).

[33] Richieri, B. *et al*. Using continuous electrical conductivity measurements to derive major solute concentrations in karst systems. *Hydrological Processes* **37**, e14929 (2023).

[34] Maréchal, J. C., Ladouche, B. & Dörfliger, N. Karst flash flooding in a Mediterranean karst, the example of Fontaine de Nîmes. *Engineering Geology* **99**, 138–146 (2008).

[35] Marechal, J.-C., Courtois, N., Jouanen, G. & Meus, P. Multi-traçage de la Fontaine de Nîmes. *karst* **56**, 1–8 (2010).

[36] Bailly-Comte, V., Ladouche, B., Charlier, J. B., Hakoun, V. & Maréchal, J. C. XLKarst, an Excel tool for time series analysis, spring recession curve analysis and classification of karst aquifers. *Hydrogeol J*10.1007/s10040-023-02710-w (2023).

[37] Fleury, P., Maréchal, J. C. & Ladouche, B. Karst flash-flood forecasting in the city of Nîmes (southern France). *Engineering Geology* **164**, 26–35 (2013).

[38] Bailly-Comte, V., Durepaire, X., Batiot-Guilhe, C. & Schnegg, P.-A. *In situ* monitoring of tracer tests: How to distinguish tracer recovery from natural background. *Hydrogeol J* **26**, 2057–2069 (2018).

[39] Blavoux, B., Mudry, J. & Puig, J.-M. Bilan, fonctionnement et protection du système karstique de la Fontaine de Vaucluse (sud-est de la France). *Geodinamica Acta* **5**, 153–172 (1992).

[40] Ollivier, C. *et al*. Karst recharge-discharge semi distributed model to assess spatial variability of flows. *Sci. Total Environ.* **703**, 134368 (2020). 10.1016/j.scitotenv.2019.134368 31731168

[41] Cholet, C. Fonctionnement hydrogéologique et processus de transport dans les aquifères karstiques du Massif du Jura. (Université de Bourgogne Franche-Comté, France, 2017).

[42] Cholet, C., Steinmann, M., Charlier, J.-B. & Denimal, S. Comparative Study of the Physicochemical Response of Two Karst Systems During Contrasting Flood Events in the French Jura Mountains. in *Hydrogeological and Environmental Investigations in Karst Systems* (eds. Andreo, B., Carrasco, F., Durán, J. J., Jiménez, P. & LaMoreaux, J. W.) 1–9, 10.1007/978-3-642-17435-3_1 (Springer Berlin Heidelberg, Berlin, Heidelberg, 2015).

[43] Klaba, V. *et al*. Multi-scale hydrostructural approach for karst environment. Application to the Arcier hydrosystem (eastern France). *Journal of Structural Geology* **184**, 105154 (2024).

[44] Klaba, V. Constraints of multiscale structural geology on hydrochemical and hydrodynamic karst models: Back to basics (Arcier, Jura, France). (Université Bourgogne Franche-Comté, 2024).

[45] Lorette, G., Lastennet, R., Peyraube, N. & Denis, A. Groundwater-flow characterization in a multilayered karst aquifer on the edge of a sedimentary basin in western France. *J. Hydrol.* **566**, 137–149 (2018).

[46] Lorette, G. *et al*. Tracing sources and fate of nitrate in multilayered karstic hydrogeological catchments using natural stable isotopic composition ( 15N-NO3- and 18O-NO3-). Application to the Toulon karst system (Dordogne, France). *J. Hydrol.* **610**, 127972 (2022).

[47] Fournier, M. *et al*. Using turbidity dynamics and geochemical variability as a tool for understanding the behavior and vulnerability of a karst aquifer. *Hydrogeol J* **15**, 689–704 (2007).

[48] Massei, N., Wang, H. Q., Dupont, J. P., Rodet, J. & Laignel, B. Assessment of direct transfer and resuspension of particles during turbid floods at a karstic spring. *Journal of Hydrology* **275**, 109–121 (2003).

[49] Massei, N., Lacroix, M., Wang, H. Q., Mahler, B. J. & Dupont, J. P. Transport of suspended solids from a karstic to an alluvial aquifer: The role of the karst/alluvium interface. *Journal of Hydrology* **260**, 88–101 (2002).

[50] Duran, L., Massei, N., Lecoq, N., Fournier, M. & Labat, D. Analyzing multi-scale hydrodynamic processes in karst with a coupled conceptual modeling and signal decomposition approach. *J. Hydrol.* **583**, 124625 (2020).

[51] Fournier, M., Massei, N., Mahler, B. J., Bakalowicz, M. & Dupont, J. P. Application of multivariate analysis to suspended matter particle size distribution in a karst aquifer. *Hydrological Processes* **22**, 2337–2345 (2008).

[52] Dausse, A., Leonardi, V. & Jourde, H. Hydraulic characterization and identification of flow-bearing structures based on multi-scale investigations applied to the Lez karst aquifer. *J. Hydrol. Reg. Stud.* **26**, 100627 (2019).

[53] Cousquer, Y. & Jourde, H. Reducing uncertainty of karst aquifer modeling with complementary hydrological observations for the sustainable management of groundwater resources. *Journal of Hydrology* **612**, 128130 (2022).

[54] Bicalho, C. C. *et al*. A conceptual model for groundwater circulation using isotopes and geochemical tracers coupled with hydrodynamics: A case study of the Lez karst system, France. *Chemical Geology* **528**, 118442 (2019).

[55] Arfib, B. & Charlier, J.-B. Insights into saline intrusion and freshwater resources in coastal karstic aquifers using a lumped Rainfall–Discharge–Salinity model (the Port-Miou brackish spring, SE France). *Journal of Hydrology* **540**, 148–161 (2016).

[56] Arfib, B. & Mocochain, L. Deep flooded karsts of south-eastern France – Genesis and hydrodynamic functioning / Karsts noyés profonds du sud-est de la France – Genèse et fonctionnement. *karst* **79**, 35–44 (2022).

[57] Zappelli, A., Arfib, B., Vallet-Coulomb, C. & Garin, T. The Freshwater Origin of a Brackish Submarine Spring Inferred from a Long-Term Stable Isotope Survey: The Port-Miou Karstic Spring (S-E France). in *Eurokarst* 2024 (eds. Fiorillo, F. et al.) 79–84, 10.1007/978-3-031-84338-9_11 (Springer Nature Switzerland, Cham, 2025).

[58] Baudement, C. *et al*. Groundwater management of a highly dynamic karst by assessing baseflow and quickflow with a rainfall-discharge model (Dardennes springs, SE France). *Bull. Soc. géol. Fr.* **188**, 40 (2017).

[59] Dufresne, C. *et al*. Karst and urban flood-induced solid discharges in Mediterranean coastal rivers: The case study of Las River (SE France). *Journal of Hydrology* **590**, 125194 (2020).

[60] Dufresne, C. *et al*. Datasets of solid and liquid discharges of an urban Mediterranean river and its karst springs (Las River, SE France). *Data in Brief* **31**, 106022 (2020). 10.1016/j.dib.2020.106022 PMC7381494 32728603

[61] Binet, S. *et al*. Water exchange, mixing and transient storage between a saturated karstic conduit and the surrounding aquifer: Groundwater flow modeling and inputs from stable water isotopes. *Journal of Hydrology* **544**, 278–289 (2017).

[62] Binet, S., Charlier, J.-B., Jozja, N., Défarge, C. & Moquet, J.-S. Evidence of long term biogeochemical interactions in carbonate weathering: The role of planktonic microorganisms and riverine bivalves in a large fluviokarst system. *Science of The Total Environment* **842**, 156823 (2022). 10.1016/j.scitotenv.2022.156823 35738376

[63] Albéric, P. River backflooding into a karst resurgence (Loiret, France). *Journal of Hydrology* **286**, 194–202 (2004).

[64] Joodi, A. S. *et al*. Development of a Darcy-Brinkman model to simulate water flow and tracer transport in a heterogeneous karstic aquifer (Val d’Orléans, France). *Hydrogeol J* **18**, 295–309 (2010).

[65] Gutierrez, A. & Binet, S. La Loire souterraine: Circulations karstiques dans le Val d’Orléans. *Géosciences* **12**, 42–53 (2010).

[66] SNO KARST. *Time series of type hydrology-hydrogeology in Le Lez (Le Salat) basin - LE BAGET (MOULIS) observatory - KARST observatory network - OZCAR Critical Zone network Research Infrastructure* 10.15148/C9CC27D4-00D0-440C-82B6-29600916A2F6 (2024).

[67] SNO KARST. *Time series of type physico-chemistry in Le Lez (Le Salat) basin - LE BAGET (MOULIS) observatory - KARST observatory network - OZCAR Critical Zone network Research Infrastructure* 10.15148/B858689A-AAAD-4E2C-8EF4-9F94E211E188 (2025).

[68] SNO KARST. *Time series of type hydrology-hydrogeology in Fontaine de Nimes basin - FONTAINE DE NIMES observatory - KARST observatory network - OZCAR Critical Zone network Research Infrastructure* 10.15148/E73A5C65-2A7D-46E5-8E27-6EDF5E452AEB (2025).

[69] SNO KARST. *Time series of type physico-chemistry in Fontaine de Nimes basin - FONTAINE DE NIMES observatory - KARST observatory network - OZCAR Critical Zone network Research Infrastructure* 10.15148/5D4514E2-61FE-46C4-AA72-316BE44FD3AE (2025).

[70] SNO KARST. *Time series of type physico-chemistry in Fontaine de Vaucluse basin - FONTAINE DE VAUCLUSE observatory - KARST observatory network - OZCAR Critical Zone network Research Infrastructure* 10.15148/FB67E0B8-4B19-4C46-875B-0E2D44EA485F (2025).

[71] SNO KARST. *Time series of type hydrology-hydrogeology in Aire d’alimentation de l’hydrosystème d’Arcier basin - JURASSIC KARST observatory - KARST observatory network - OZCAR Critical Zone network Research Infrastructure* 10.15148/792C2468-D839-4AD3-8EF0-B928A6656E8C (2025).

[72] SNO KARST. *Time series of type physico-chemistry in Aire d’alimentation de l’hydrosystème d’Arcier basin - JURASSIC KARST observatory - KARST observatory network - OZCAR Critical Zone network Research Infrastructure* 10.15148/BDFFFA66-860C-4245-A11A-4FBBABC521E3 (2025).

[73] SNO KARST. *Time series of type hydrology-hydrogeology in Le Doubs basin - JURASSIC KARST observatory - KARST observatory network - OZCAR Critical Zone network Research Infrastructure* 10.15148/3B65D490-0AEB-4B30-84A5-C5D46CFA855B (2025).

[74] SNO KARST. *Time series of type physico-chemistry in Le Doubs basin - JURASSIC KARST observatory - KARST observatory network - OZCAR Critical Zone network Research Infrastructure* 10.15148/09929CF0-6A6B-414E-B5BD-25A9B8C78D88 (2025).

[75] SNO KARST. *Time series of type chemistry in Le Doubs basin - JURASSIC KARST observatory - KARST observatory network - OZCAR Critical Zone network Research Infrastructure* 10.15148/841D3677-FDDC-454C-8A71-ABAC2851ED6F (2025).

[76] SNO KARST. *Time series of type hydrology-hydrogeology in Loue-Lison basin - JURASSIC KARST observatory - KARST observatory network - OZCAR Critical Zone network Research Infrastructure* 10.15148/A2CA20F9-CD78-4260-84D4-CEB4B1426666 (2025).

[77] SNO KARST. *Time series of type physico-chemistry in Loue-Lison basin - JURASSIC KARST observatory - KARST observatory network - OZCAR Critical Zone network Research Infrastructure* 10.15148/5EBB9D5F-081A-405A-8290-AD29A084E4E9 (2025).

[78] SNO KARST. *Time series of type chemistry in Loue-Lison basin - JURASSIC KARST observatory - KARST observatory network - OZCAR Critical Zone network Research Infrastructure* 10.15148/A815F209-0CA1-4240-89FD-E0D0418A6E5D (2025).

[79] SNO KARST. *Time series of type hydrology-hydrogeology in L’Isle basin - KARSTS AQUITAINS observatory - KARST observatory network - OZCAR Critical Zone network Research Infrastructure* 10.15148/FCF00F41-F22C-47C0-BEB4-031255793259 (2025).

[80] SNO KARST. *Time series of type physico-chemistry in L’Isle basin - KARSTS AQUITAINS observatory - KARST observatory network - OZCAR Critical Zone network Research Infrastructure* 10.15148/6C7A0DB4-2515-4488-9E70-94CC4454FA8A (2025).

[81] SNO KARST. *Time series of type hydrology-hydrogeology in Le Theluet basin - KARST DE LA CRAIE observatory - KARST observatory network - OZCAR Critical Zone network Research Infrastructure* 10.15148/15AD51CD-784C-4EF7-B547-BD3C620C0512 (2025).

[82] SNO KARST. *Time series of type physico-chemistry in Le Theluet basin - KARST DE LA CRAIE observatory - KARST observatory network - OZCAR Critical Zone network Research Infrastructure* 10.15148/E4D0AD5D-EAAB-4F9D-BAF4-B34F078F89C0 (2025).

[83] SNO KARST. *Time series of type hydrology-hydrogeology in Le Lez (Méditerranée) basin - MEDYCYSS observatory - KARST observatory network - OZCAR Critical Zone network Research Infrastructure* 10.15148/CFD01A5B-B7FD-41AA-8884-84DBDDAC767E (2025).

[84] SNO KARST. *Time series of type physico-chemistry in Le Lez (Méditerranée) basin - MEDYCYSS observatory - KARST observatory network - OZCAR Critical Zone network Research Infrastructure* 10.15148/38BD5FA8-A7E1-4900-A1F6-2D59C3F31DFE (2025).

[85] SNO KARST. *Time series of type hydrology-hydrogeology in Port-Miou basin - PORT-MIOU PROVENCE observatory - KARST observatory network - OZCAR Critical Zone network Research Infrastructure* 10.15148/152EA7A3-A754-4248-BD5D-2BFEC1BFF17D (2025).

[86] SNO KARST. *Time series of type physico-chemistry in Port-Miou basin - PORT-MIOU PROVENCE observatory - KARST observatory network - OZCAR Critical Zone network Research Infrastructure* 10.15148/A757D9F3-49F4-42A4-80C6-2A7C423FA156 (2025).

[87] SNO KARST. *Time series of type hydrology-hydrogeology in Dardennes - Le Las basin - PORT-MIOU PROVENCE observatory - KARST observatory network - OZCAR Critical Zone network Research Infrastructure* 10.15148/E3FCFEB1-E683-4BFE-B5E8-717DD04E2FB4 (2025).

[88] SNO KARST. *Time series of type physico-chemistry in Dardennes - Le Las basin - PORT-MIOU PROVENCE observatory - KARST observatory network - OZCAR Critical Zone network Research Infrastructure* 10.15148/18B4D8BF-AA12-4159-92FC-23EC70D1198E (2025).

[89] SNO KARST. *Time series of type hydrology-hydrogeology in La Loire basin - VAL D’ORLEANS observatory - KARST observatory network - OZCAR Critical Zone network Research Infrastructure* 10.15148/EC4D084C-ED89-4CD0-946A-076BF166A150 (2025).

[90] SNO KARST. *Time series of type physico-chemistry in La Loire basin - VAL D’ORLEANS observatory - KARST observatory network - OZCAR Critical Zone network Research Infrastructure* 10.15148/CDF4F839-D6A8-4047-86FC-4F81B2295DB6 (2025).

[91] Mazzilli, N. *et al*. KarstMod: A modelling platform for rainfall - discharge analysis and modelling dedicated to karst systems. *Environ. Model. Softw.* **122**, 103927 (2019).

[92] Sivelle, V. *et al*. Improvement of the KarstMod modelling platform for a better assessment of karst groundwater resources. *Hydrol. Earth Syst. Sci.* **29**, 1259–1276 (2025).

[93] Cinkus, G., Mazzilli, N. & Jourde, H. KarstID: An R Shiny application for the analysis of karst spring discharge time series and the classification of karst system hydrological functioning. *Environ Earth Sci* **82**, 6 (2023).

[94] Sarrazin, F., Hartmann, A., Pianosi, F., Rosolem, R. & Wagener, T. V2Karst V1.1: A parsimonious large-scale integrated vegetation–recharge model to simulate the impact of climate and land cover change in karst regions. *Geosci. Model Dev.* **11**, 4933–4964 (2018).

[95] Bittner, D., Narany, T. S., Kohl, B., Disse, M. & Chiogna, G. Modeling the hydrological impact of land use change in a dolomite-dominated karst system. *Journal of Hydrology* **567**, 267–279 (2018).

